# Effect of different preventive agents on bracket shear bond strength: *in vitro study*

**DOI:** 10.1186/1472-6831-14-28

**Published:** 2014-03-29

**Authors:** Huda M Al-Kawari, Asma M Al-Jobair

**Affiliations:** 1 Department of Pediatric Dentistry and Orthodontics, College of Dentistry, King Saud University, Riyadh, Saudi Arabia

**Keywords:** Orthodontic brackets, Shear bond strength, CPP-ACP paste, Fluoride-containing-CPP-ACP, 5% Sodium fluoride

## Abstract

**Background:**

The effects of fluoride and CPP-ACP before bracket bonding on the shear bond strength of orthodontic brackets have been reported with contradicting results. The objective of this *in vitro* study was to evaluate the effect of different preventive agents namely*;* casein phosphopeptide-amorphous-calcium-phosphate (CPP-ACP), fluoride-containing-CPP-ACP (CPP-ACPF) and 5% sodium fluoride (5% NaF), on the enamel-bracket shear bond strength (SBS) and to compare their effects when applied before or after acid-etching.

**Methods:**

Human premolar teeth were randomly divided into seven groups (16 teeth per group) as follows: the control group, where no preventive agent was applied on the enamel and 6 experimental groups. Teeth in groups 1a, 2a, and 3a were treated with CPP-ACP paste, CPP-ACPF paste, and 5% NaF, respectively before acid-etching. Teeth in groups 1b, 2b and 3b were treated using the same preventive agents after acid-etching. The brackets were then bonded and the teeth were thermocycled. The brackets' SBS was measured and the adhesive remnant was assessed using adhesive remnant index (ARI). Analysis of variance (ANOVA) and Tukey test were performed to compare the SBS among different groups. Chi-square test was used to evaluate differences in ARI scores between the groups.

**Results:**

Enamel surface treatment with CPP-ACPF after acid-etching significantly increased SBS compared to the control and to its application before acid-etching (P < 0.05). Higher ARI index was recorded when the preventive agents were applied after acid-etching.

**Conclusion:**

Brackets' SBS significantly increased when fluoride-containing-CPP-ACP was applied after acid-etching.

## Background

Orthodontic treatment complicates oral hygiene maintenance due to the presence of brackets, bands, springs, coils and arch wires [1]. In addition, acid-etching might trim down 5–10 μm of the enamel surface, which can result in a permanent demineralization of enamel surface beneath or adjacent to the bracket [2]. Moreover, excess bonding material around the bracket base creates sites where dental plaque accumulation is easier [1]. All these factors subsequently increase the risk of initial caries lesions during orthodontic therapy. It has been reported that half of the patients who receive fixed orthodontic treatment develop white spot lesions [3]. Therefore, enamel surface treatment with different caries preventive agents prior and/or during orthodontic treatment has been suggested.

Fluoride is widely used for caries prevention; its incorporation into the enamel results in a surface less soluble in an acid environment [4]. Fluoride ions prevent demineralization and promote remineralization. This is attributed to the fluoride-enhanced precipitation of calcium phosphates, and the formation of fluorhydroxyapatite in the dental tissues [4]. The acidic dental materials such as etchants used in bracket bonding in orthodontic treatments might increase the caries rate [2]. Therefore, fluoride could be used to reduce this iatrogenic enamel damage, when applied prior to bracket bonding. However, enamel surface treatment with fluoride might affect the bond strength of bonded brackets and might lead to bracket failure. Several methods of topical fluoride application have been proposed at different steps of orthodontic bracket bonding procedure. The effects of fluoride application methods before acid-etching [5-7], during acid-etching when the fluoride is added to acid etchant [8,9], and after acid-etching [10,11] on bond strength of orthodontic brackets have been previously investigated with controversial results. Some investigations showed significant reduction in bond strength values when fluoride was applied before [5,12] or after acid-etching [13,14]. On the contrary, other studies demonstrated that the topical application of fluoride to enamel surfaces did not negatively influence the bracket bond strength before [6,15] or after [10,11] acid-etching.

In addition to fluoride, a casein phosphopeptide-amorphous calcium phosphate (CPP-ACP) and fluoride-containing-CPP-ACP pastes have also been recommended for caries prevention and enamel remineralization [16-18]. It involves the incorporation of the nano-complexes into dental plaque and onto the tooth surface, thereby acting as a calcium and phosphate reservoir [16]. Topically administered CPP-ACP buffers free calcium and phosphate ion activity, maintaining a state of supersaturation with respect to tooth enamel which in turn helps in preventing demineralization and facilitates remineralization [19]. Therefore, the application of CPP-ACP preparations for prevention and treatment of initial caries lesions in orthodontics has been projected [16,20,21]. However, similar to fluoride, enamel treated with topical application of CPP-ACP has been shown to be more resistant to subsequent acid-etching [20], and consequently may lead to brackets failure. Contradicting results have been reported recently concerning the effects of CPP-ACP preparations on the bond strength of orthodontic brackets. Some studies showed significant reduction in bond strength values when CPP-ACP agents were applied before acid-etching [22,23]. However, other studies revealed that the topical application of CPP-ACP agents to enamel surfaces before acid-etching did not harmfully influence the bracket bond strength [12,24-27]. Conversely, significant increase in shear bond strength was reported after application of CPP-ACP before acid-etching process [28].

Nevertheless, in all of the aforementioned investigations, CPP-ACP agents were applied before acid-etching. However, the application of these agents after acid-etching may present preventive effect without jeopardizing the bond strength of orthodontic brackets. Therefore, the effect of their application after acid-etching needs to be investigated. Consequently, the objective of this study was to evaluate *in vitro* the effect of different preventive agents namely*;* casein phosphopeptide-amorphous-calcium-phosphate (CPP-ACP), fluoride-containing-CPP-ACP (CPP-ACPF) and 5% sodium fluoride (5% NaF), on the enamel-bracket shear bond strength (SBS) and to compare their effects when applied before or after acid-etching.

## Methods

After the approval of the College of Dentistry Research Centre (CDRC) at King Saud University for this study, a sample of 112 extracted human premolar teeth were collected following patients’ verbal consent to include their teeth in the study. All teeth were examined for cracks or/and caries then were cleaned with brush and pumice and stored in physiological saline. The teeth were randomly divided into seven groups (16 teeth/group), one control and six experimental groups. The agents for enamel surface treatment and time for acid-etching for all groups are listed in Table 1.

**Table 1:** Preventive agents tested and time of acid-etching for all groups

**Preventive agents**	**Group no.**	**No. of teeth**	**Before acid-etching**	**After acid-etching**
**Control**	C	16	No treatment	No treatment
**CPP–ACP paste**	1a	16	CPP–ACP paste	No treatment
**(MI paste)**	1b	16	No treatment	CPP–ACP Paste
**CPP–ACPF paste**	2a	16	CPP–ACPF paste	No treatment
**(MI paste plus)**	2b	16	No treatment	CPP–ACPF paste
**5% NaF varnish**	3a	16	5% NaF varnish	No treatment
**(Fluoraphat)**	3b	16	No treatment	5% NaF varnish

CPP-ACP: casein phosphopeptide–amorphous-calcium-phosphate, CPP–ACPF: fluoride-containing CPP–ACP, NaF: Sodium fluoride.

### Control group

Enamel surfaces were acid-etched according to the manufacturers’ instructions with 35% phosphoric acid (Ultra Etch, Ultradent Products, INC) for 30 s, rinsed with water spray for 15 s, air-dried until desiccated.

### Experimental groups

#### Group 1a

Enamel surfaces were pre-treated with CPP-ACP paste (MI Paste, GC Corporation, Tokyo, Japan) for 3 min [12], left undisturbed for 30 min [7], then rinsed and dried then acid-etched as control group.

#### Group 1b

Enamel surfaces were first acid-etched then subjected to the same agent used in group 1a.

#### Group 2a

Enamel surfaces were pre-treated with CPP-ACPF paste (MI Paste Plus, GC Corporation, Tokyo, Japan) for 3 min, left undisturbed for 30 min [7], then rinsed and dried then acid-etched as control group.

#### Group 2b

Enamel surfaces were first acid-etched then subjected to the same agent used in group 2a.

#### Group 3a

Enamel surfaces were pre-treated with 5% NaF (Fluoraphat pro, PROMEDICA, Germany) for 4 min [12], left undisturbed for 3 min [7], then rinsed and dried then acid-etched as control group.

#### Group 3b

Enamel surfaces were first acid-etched and then subjected to the same agent used in group 3a.

The bonding process was carried out according to the manufacturers’ instructions and guidelines. Primer (Transbond™ XT primer, 3 M Unitek, USA) was applied on acid-etched enamel and on the bracket base (Mini Dimond Twin, Ormco, USA). Transbond adhesive (Transbond™ XT/LR, 3 M Unitek, USA) was then applied on bracket base, and bracket was positioned on the center of the buccal surface of the crown then light-cured (3 M EPSE, Elipar-510 Curing Light). Each bracket was subjected to 20 s light curing applied from both mesial and distal sides. Specimens were then stored in distilled water at 37°C for 24 h, and then were thermocycled (Thermocycler THE 1100/1200, Huber, Germany) in distilled water at 5 ± 2°C-55 ± 2°C for 1000 cycles with a dwell time of 30 s and a transfer time of 10 s.

### Shear bond strength test

All teeth were embedded in acrylic resin using mounting jig to align the buccal surface of each tooth perpendicular to the bottom of the mold. The specimens were then mounted in the jig, which was attached to a universal Inestron testing device (Model-1197, Inestron, UK). The specimens were secured in the lower jaw of the machine and the bracket base was paralleled to the direction of the shear force. Continuous shear force was applied as close as possible to the tooth/bracket interface with a sharp chisel-shaped rod attached to the end of the Inestron machine at a crosshead speed of 1 mm per minute until bracket detached.

After brackets were detached, each tooth surface was examined under a stereo-microscope (SMZ-1000, Nikon Instruments Inc., Japan) at 10× magnification to assess the amount of adhesive remnants using the adhesive remnant index (ARI) system [29]. The adhesive remnant was coded and recorded by an external examiner unaware of the study. The amount of adhesive remnant on the tooth surface was ranked from 0-3 as follows; zero = no adhesive remnant; 1 = less than 50% adhesive remnant; 2 = more than 50% adhesive remnant and 3 = 100% adhesive remnant. The intra-examiner reliability for ARI scoring was determined by the same examiner re-examining 15 teeth, one week after the first examination.

### Statistical analysis

Statistical analysis was performed using the Statistical Package for Social Sciences 16 software (SPSS Inc., Chicago, Ill). The data were analyzed using analysis of variance (ANOVA) and Tukey multiple comparison test. The ARI scores were analyzed using chi-square test. P value less than 0.05 was considered statistically significant.

## Results

Two-way ANOVA showed that shear bond strength of orthodontic bracket was significantly impacted by the application of preventive agents as well as by the time of acid-etching (Table 2). However, there was no interaction between the two factors. Descriptive statistics (including mean and standard deviation) and the results of one-way ANOVA and the post-hoc Tukey test comparing the SBS values between control and experimental groups are presented in Table 3. A one-way ANOVA indicated that there was a significant difference in the shear bond values among the different groups (P < 0.000) (Table 3). According to Tukey's multiple comparisons, significant difference was detected between the control and CPP-ACPF group after acid-etching (control and group 2b) (P < 0.05). However, within the same agent groups, significant difference was found between the applications of CPP-ACPF before and after acid-etching (groups 2a and 2b) (P < 0.05). In all experimental groups, higher shear bond strength values were noticed when the preventive agents were applied after acid-etching. In addition, 5% NaF groups showed the lowest SBS values compared to CPP-ACP and CPP-ACPF groups whether applied before or after acid-etching.

**Table 2:** Results from two-way ANOVA test investigating the effects of the two explanatory factors (Preventive agents, and the time of acid-etching), and the interaction between them on SBS

	**Mean square**	**F**	**P**
**Preventive agents groups**	128.143	6.103	0.003
**Time of acid-etching (before or after acid-etching groups)**	339.524	16.169	0.000
**Preventive agents X Time of acid-etching**	18.222	0.868	0.423

**Table 3:** Results from one-way ANOVA and the Tukey test comparing the SBS values between control and experimental groups

**Time of acid-etching**	**Preventive agents**				**ANOVA**
	**Control**	**CPP–ACP MI paste (groups 3)**	**CPP–ACPF MI paste plus (groups 2)**	**5% NaF fluoraphat varnish (groups 3)**	
			Mean (standard deviation)		
**Before (groups a)**	11.25 (4.27)^a,b*^	13.37 (4.79)^a,b,c^	11.05 (4.85)^a,b^	8.86 (4.35)^a^	0.000
**After (groups b)**		15.65 (5.87)^b,c^	16.35 (3.81)^c^	12.56 (3.74)^a,b,c^	

^*^Tukey test: Different letters indicate significant differences (P < 0.05), CPP-ACP: casein phosphopeptide–amorphous-calcium-phosphate, CPP–ACPF: fluoride-containing CPP–ACP, NaF: Sodium fluoride.

Table 4 presents the findings of the assessment of the adhesive remnants on the teeth surfaces. The Chi-square test indicated statistical difference in ARI scores among groups (P = 0.034). Generally, more composite remnants on teeth surfaces were found when the preventive agents were applied after acid-etching (Table 4). This was characterized by a shift from ARI scores of 0 and 1 before acid-etching to ARI scores of 2 and 3 after acid-etching. However, within the same agent, significant difference in the ARI scores was only detected between 5% NaF groups (groups 3a and 3b) (P = 0.016). The intra-examiner reliability for ARI measurements indicated high value (0.92) using Kappa test.

**Table 4:** Frequency of distribution of Adhesive Remnant Index (ARI)^* ^**scores and chi-square comparison between the groups**

**Groups (16 teeth/group)**		**ARI scores no. (%)**				**P^**	**P^^**
		**0**	**1**	**2**	**3**		
**Control group no treatment**	Control	1 (6.3)	5 (31.3)	7 (43.8)	3 (18.8)	0.034	
**CPP–ACP groups**	Group 1a	0 (0)	7 (43.8)	6 (37.5)	3 (18.8)		0.311
	Before acid-etch						
**MI Paste**	Group 1b	0 (0)	5 (31.3)	4 (25)	7 (43.8)		
	After acid-etch						
**CPP–ACPF groups**	Group 2a	4 (25)	6 (37.5)	5 (31.3)	1 (6.3)		0.175
	Before acid-etch						
**MI paste plus**	Group 2b	1 (6.3)	4 (25)	6 (37.5)	5 (31.3)		
	After acid-etch						
**5% NaF groups**	Group 3a	4 (25)	10 (62.5)	1 (6.3)	1 (6.3)		0.016
	Before acid-etch						
**Fluoraphat varnish**	Group 3b	0 (0)	6 (37.5)	6 (37.5)	4 (25)		
	After acid-etch						

^*^ARI Scores = 0- no adhesive remaining on the enamel surface; 1- less than 50% adhesive remaining on tooth; 2- more than 50% adhesive remaining on tooth; and 3- all adhesive remaining on tooth surface.

^P = among all groups ^^P = within the same agent groups before and after acid-etch.

## Discussion

Orthodontic patients are often at high risk of developing dental caries during orthodontic treatment, especially when their compliance to oral hygiene instructions is doubtful [30]. Many preventive protocols have been suggested to overcome this problem such as using fluoride releasing adhesives and the use of preventive agents on enamel surface before bracket bonding [13,22,31].

Acid etchant demineralises the hydroxyapatite crystals of enamel rods, and exposes micropores on the enamel allowing the adhesive material to interlock. Thus, the bracket might resist the masticatory forces [2]. Fluoride and Casein phosphopeptide-amorphous-calcium-phosphate are delivering systems that allow freely available fluoride, calcium and phosphate ions to enter enamel and reform into stronger crystals which in turn help in preventing demineralization and enhance reminralization [4,16-18]. This mechanism may interfere with acid-etching process.

In the present study, shear bond strength of orthodontic bracket was significantly impacted by the application of preventive agents as well as by the time of acid-etching, which means that both factors have almost the same impact on SBS. The SBS increased significantly when CPP-ACPF was applied after acid-etching. To our knowledge, there are no published studies of the effects of CPP-ACPF or CPP-ACP after acid-etching process on bracket SBS. Nevertheless, in all experimental groups, the bracket SBS values increased when the preventive agents were applied after acid-etching. This might be attributed to the fact that when CPP-ACP, CPP-ACPF and fluoride were applied before acid-etching, they increase the resistance of tooth enamel to acid, which can affect bracket adhesion and result in lowering the bracket SBS values [32,33]. Teeth with a higher concentration of fluoride are generally considered to be more resistant to acid-etching and need a longer etching time [34]. Moreover, enamel lesions which have been remineralised as a result of topical exposure to CPP-ACP showed more resistant to subsequent acid etching [32]. Hirce et al. [35] supported the use of topical fluoride after acid-etching, a procedure that achieves the benefits of increased fluoride uptake without changing the bond strength of the resin adhesive. Although, application of preventive agents after acid-etching resulted in higher SBS value than when applied before acid-etching, there was no significant difference in the SBS between the control and the other experimental groups when applied before acid-etching. This indicated that the application of these preventive agents before acid-etching has no adverse effect on bracket bonding strength. This was in accordance with Xiaojun et al. [18] who found that the use of CPP-ACP before acid-etching did not compromise the bracket bond strength. Tabrizi and Cakirer [12] found also that application of CPP-ACP, either alone or combined with fluoride before acid-etching may safely be used as a prophylactic agent prior to bracket bonding. In addition, Park et al. [24] reached a similar conclusion regardless of the adhesive system used for bond brackets on the teeth. Furthermore, Çehreli et al. [25] reported that the application of CPP-ACPF before acid-etching has no effect on bracket SBS when using self-etching systems, but when applying its non-fluoride version CPP-ACP, significant decrease in bracket SBS was evident. Contradictory to our result, Kecik et al. [28] observed that the application of CPP-ACP alone or combined with acidulated phosphate fluoride (APF) has shown significant increase in the SBS of orthodontic brackets.

The use of fluoride during different step of orthodontic bracketing is still debatable due to its effect on SBS of orthodontic brackets [6-13]. Cacciafesta et al. [13] showed significantly lower SBS with fluoride application before or after acid-etching. Tabrizi and Cakirer [12] found that application of 5% NaF alone before acid-etching was found to decrease bracket SBS. However, Kimura et al. [15] reported that there was no difference in bond strength of orthodontic brackets in enamel treated either with fluoride varnish or not, when applied before acid-etching. In the present study, although no statistical difference was found between the control group and the 5% NaF group 3a when applied before acid-etching, the lowest SBS value was recorded in this group. This might be explained by that fluoride reacts with the enamel surface to form calcium-fluoride and fluorapatite which make the enamel more resistant to demineralization effect of acid etchant and consequently reduce bond strength by disrupting the formation of enamel tags [33].

In the current study, 5% NaF groups 3a and 3b showed the lowest SBS values compared to CPP-ACP and CPP-ACPF groups. This might be related to the differences in their reaction with enamel surface and their remineralisation mechanism. It has been shown that enamel remineralised with fluoride was harder than enamel treated with CPP-ACP [36]. In addition, application of a higher concentration of sodium fluoride may justify the lower bond strength in 5% NaF groups, before or after acid-etching. Khosravanifard et al. [37] found that the proportion of additional fluoride correlated significantly and negatively with the bond strength of orthodontic bracket. Furthermore, the differences in the physical properties between the varnish and the paste might have some effects. The varnish evaporated quickly to form a thin film on surface, while the paste might be lost after washing [36].

The ARI scores indicated that more composite remnant on the teeth when the preventive agents were applied after acid-etching as bond failure frequently occurred at the bracket-adhesive interface. This was characterized by a shift from ARI scores of 0 and 1 before acid-etching to ARI scores of 2 and 3 after acid-etching. In the present study and others [15,38,39], the distribution of ARI scores were in accordance with the SBS values of the groups. This means that enamel surface treatment with preventive agent after acid-etching does not compromise the bonding of the resin to the enamel, although, it is clinically not advantageous. According to the adhesive remnant index (ARI) system used in this study [29], the most desired clinical condition is a low ARI score with less composite remaining on the enamel surface. Therefore, the possibility of enamel damage after debonding decreases, as less adhesive remnant on the tooth has been advocated for easier and safer removal of residual adhesive.

The results of this study should be carefully compared to others findings due to the differences in methodology, such as teeth selection, selection of preventive agents, application time, fluoride concentrations, bonding system and thermocycling.

However, within the limitations of this *in vitro* study, it can be anticipated that enamel surface treatment with preventive agents before or after acid-etching in orthodontic bracket bonding would be beneficial in poorly compliant patients to prevent the initiation of carious lesion without disturbing bonding strength. The use of CPP-ACPF preventive agent after acid-etching is recommended as it provided the maximum value in brackets' SBS. The use of this agent probably decreases the incidence of initial caries lesion as reported by many investigators due to the synergistic effect of both the CPP-ACP and NaF [17,18]. Further clinical studies to investigate the clinical effectiveness of preventive agents before orthodontic bracket bonding would be required to prove these *in vitro* results.

## Conclusion

Enamel surface treatment with fluoride-containing-CPP-ACP after acid-etching resulted in significant increase in SBS value of orthodontic brackets. In general, higher shear bond strength values were achieved when the preventive agents were applied after acid-etching. However, their application before acid-etching process does not compromise bracket SBS.

## Competing interests

The authors declare that they have no competing interests.

## Authors’ contributions

HMK and AMJ contributed to the design, data collection, analysis and write-up and finalized the manuscript. Both authors read and approved the final manuscript.

## Pre-publication history

The pre-publication history for this paper can be accessed here:

http://www.biomedcentral.com/1472-6831/14/28/prepub
